# Molecular signatures in Mendelian neurodevelopment: a focus on ubiquitination driven DNA methylation aberrations

**DOI:** 10.3389/fnmol.2024.1446686

**Published:** 2024-07-29

**Authors:** Liselot van der Laan, Nicky ten Voorde, Marcel M. A. M. Mannens, Peter Henneman

**Affiliations:** ^1^Department of Human Genetics, Amsterdam UMC, Amsterdam, Netherlands; ^2^Amsterdam Reproduction and Development, Amsterdam University Medical Centers, University of Amsterdam, Amsterdam, Netherlands

**Keywords:** ubiquitination pathway, TRIP12, USP7, DNA methylation, epigenetic machinery, Mendelian disorders

## Abstract

Mendelian disorders, arising from pathogenic variations within single genetic loci, often manifest as neurodevelopmental disorders (NDDs), affecting a significant portion of the pediatric population worldwide. These disorders are marked by atypical brain development, intellectual disabilities, and various associated phenotypic traits. Genetic testing aids in clinical diagnoses, but inconclusive results can prolong confirmation processes. Recent focus on epigenetic dysregulation has led to the discovery of DNA methylation signatures, or episignatures, associated with NDDs, accelerating diagnostic precision. Notably, TRIP12 and USP7, genes involved in the ubiquitination pathway, exhibit specific episignatures. Understanding the roles of these genes within the ubiquitination pathway sheds light on their potential influence on episignature formation. While TRIP12 acts as an E3 ligase, USP7 functions as a deubiquitinase, presenting contrasting roles within ubiquitination. Comparison of phenotypic traits in patients with pathogenic variations in these genes reveals both distinctions and commonalities, offering insights into underlying pathophysiological mechanisms. This review contextualizes the roles of TRIP12 and USP7 within the ubiquitination pathway, their influence on episignature formation, and the potential implications for NDD pathogenesis. Understanding these intricate relationships may unveil novel therapeutic targets and diagnostic strategies for NDDs.

## Introduction

Mendelian disorders arise from pathogenic variations occurring within a single genetic locus and are frequently associated with neurodevelopmental disorders (NDDs), which impact over 3% of children worldwide (Marrus and Hall, 2017; Acharya et al., 2022). NDDs primarily involve dominant and somatic genetic variants, and infants born with such conditions experience atypical brain and central nervous system development, that are associated with distinct types of intellectual disabilities (ID). Moreover, other commonly observed traits in children with NDDs include disruptions in normal growth, limb anomalies, distinctive facial features, and macrocephaly (Bjornsson, 2015). Presently, various genetic tests are employed to confirm clinical diagnoses of individuals with NDDs. However, in numerous cases, inconclusive genetic tests may stress and extend the turnaround time for molecular confirmation significantly. Intriguingly, several of these Mendelian disorders are linked to dysfunctions within the epigenetic machinery (Fahrner and Bjornsson, 2019). The latter concept led to the discovery of hundreds of specific and selective DNA methylation signatures. The genome diagnostic implementation of these newly discovered DNA methylation, or so-called episignatures, accelerated molecular confirmation of aforementioned inconclusive results (Sadikovic et al., 2019). Meaning, that instead of initially searching for a specific variant, diagnostic labs can first identify and match a highly accurate episignature profile that confirms a specific type of NDD. Following this, sequencing methods such as whole exome sequencing (WES) can be used to pinpoint the specific variants associated with the identified episignature (Bjornsson, 2015; Haghshenas et al., 2020; Levy et al., 2022). Episignatures have become a powerful tool in the clinical diagnostic setting, enabling the identification and confirmation of genetic disorders with high precision. These DNA methylation patterns provide a unique biomarker for various NDDs and can be used to guide genetic testing and patient management. An recent article by Kerkhof et al. (2024) demonstrates the diagnostic utility in genetically undiagnosed rare diseases. Recently we reported on specific DNA methylation signatures of two of these NDD linked genes, both implicated in the ubiquitination pathway, namely: the *TRIP12* gene encoding for Thyroid Hormone Receptor Interactor 12 protein and the *USP7* gene, encoding for the Ubiquitin Specific Peptidase 7 protein (van der Laan et al., 2022a, 2023). The mutations in USP7 and TRIP12 are hypothesized to influence episignatures due to their critical roles in epigenetic regulation. USP7 is involved in deubiquitination processes that affect the stability and function of various proteins, including those involved in chromatin remodeling and DNA methylation. Similarly, TRIP12, as a HECT-type E3 ubiquitin ligase, regulates protein degradation pathways that are essential for maintaining cellular homeostasis, including those impacting epigenetic modifications. These proteins interact with components of the epigenetic machinery, such as histone modifiers and DNA methyltransferases, influencing chromatin structure and gene expression. Therefore, mutations disrupting USP7 and TRIP12 function can lead to dysregulated epigenetic processes, resulting in altered DNA methylation patterns characteristic of episignatures associated with neurodevelopmental disorders and other conditions (van der Laan et al., 2022a,b, 2023).

Epigenetic modifications refer to reversible and mitotically heritable changes in the genome without altering the DNA sequence itself. Epigenetics plays a crucial role in the regulation of gene expression, making it essential during early development. Epigenetic features comprise histone modifications, DNA methylation, and regulatory microRNAs. In particular, histone and DNA modifications result in either more accessible or more closed chromatin structure. The most extensively studied epigenetic feature is DNA methylation, which involves the addition of a methyl group to the cytosine of a CpG dinucleotide (Younesian et al., 2022). Histone modifications, however, are more complex and involve covalent alterations—such as acetylation, phosphorylation, methylation, and ubiquitination—added to specific positions of particular histone tails. DNA and histone modification have been shown to be highly associated. The third epigenetic feature comprises non-coding RNAs that regulate gene expression more directly, i.e., by interacting with mRNA (Ruffo et al., 2023). The proteins engaged in the epigenetic machinery can be categorized into four subclasses: writers, erasers, readers, and remodelers. Writers are responsible for adding modifications to the genome, dependent on the cell type, developmental stage, and metabolic state of the cell. Erasers have the capacity to remove these marks, while readers interpret them. Within the realm of modifying histone tails, chromatin remodelers play a role by moving nucleosomes along DNA, thereby altering the accessibility of that DNA (Fahrner and Bjornsson, 2014). Numerous previously reported studies described over 100 distinct NDD linked DNA methylation signatures, for which the majority involved a pathogenic variant in genes encoding a protein involved in the epigenetic machinery (van der Laan et al., 2022b).

This relatively large number of discovered DNA methylation signatures raises the possibility of overlap in differentially methylated positions (DMPs) among various episignatures, potentially contributing to the emergence of functionally interconnected entities (Levy et al., 2022). For example, such intricate interplay becomes evident in the episignature associated with *TRIP12*, which exhibits partial overlap of DMPs included in the signature linked to BAFopathies (*ARID1B; ARID1A; SMARCB1; SMARCA4; SMARCA2*) (van der Laan et al., 2022a). BAFopathies encompass a group of syndromes resulting from dysfunction in the BRG1-Associated Factor (BAF) protein complex, a key chromatin remodeling complex. Studies have indeed demonstrated the interaction between TRIP12 and BAF57 proteins, the latter being a constituent protein of the BAF complex (Keppler and Archer, 2010). However, the generally observed overlap between episignatures associated with pathogenic variation in different genes may not, *per se*, imply direct involvement of the exact same molecular pathway, since consequential epigenetic effects may also encompass partially similar probe sets. On the other hand, distinct pathogenic variations within a single gene may not consistently yield identical episignatures (Sadikovic et al., 2019, 2021). For example, different types of pathogenic variants within the lysine acetyltransferase 6B (*KAT6B*) gene are linked to two distinct syndromes, namely genitopatellar syndrome (GPS) or Say-Barber-Biesecker-Yong-Simpson syndrome (SBBYSS). These two distinct syndromes are associated with two different episignatures as well (Levy et al., 2022). Altogether, it can be stated that NDDs and their signatures are highly complex, meaning that they can intergenetically exhibit a substantial signature overlap, or intergenetically, may conceal signature overlap.

The proteins encoded by the *TRIP12* and *USP7* genes both play crucial roles in the ubiquitination pathway. Ubiquitination involves attaching one or more ubiquitin proteins to target proteins, marking them for degradation by the proteasome, influencing their cellular localization or drive interaction with other molecules (Rape, 2018). While it is understood that dysfunction within the ubiquitination pathway can contribute to various diseases, including neurodegenerative disorders (van der Laan et al., 2022a), much remains unknown regarding the exact pathological molecular mechanisms. Within this context, there is a lack of understanding about how genetic variants in genes related to the ubiquitination pathway lead to diverse DNA methylation patterns.

Proteins with Methyl-CpG-Specific Binding Domains (MBDs), like MECP1 and MECP2, bind to DNA methylation and recruit histone modifying enzymes, repressing transcription and forming complexes with factors like lysine deacetylases and H3K9 methyltransferases (Jeltsch et al., 2018). MECP2 also acts as a gene activator, illustrating its dual role in neurodevelopment and its association with RETT syndrome (Rajavelu et al., 2018). Such interplay represents an example of a direct link between histone modifications, DNA methylation, and neurodevelopmental disorders. Hence, pathogenic variants within the *MECP2* gene have been associated with RETT syndrome, characterized by symptoms such as learning disabilities, repetitive stereotyped hand movements, and developmental regression (Liyanage and Rastegar, 2014). Proteins with a CXXC domain, such as MECP1, bind to unmethylated regulatory regions, although MECP2’s specific binding mechanism remains unclear (Li et al., 2015; Lei et al., 2019). Other proteins like KDM2A, KMT2A/B, H3K4-specific methyltransferases, TET1, and TET3 maintain unmethylated CG islands through their CXXC domains (Jeltsch et al., 2018). In essence, the diverse assortment of proteins discussed highlights the intricate regulatory mechanisms involved in maintaining the unmethylated state of DNA regions, emphasizing the multifaceted nature of DNA methylation regulation.

Bidirectional interactions between DNA methylation and histone modifications are evident; for example, H3K36 methylation recruits DNMT3A for DNA methylation, while H3K4 methylation can impede DNMT3 binding (Loaeza-Loaeza et al., 2020; Zhang et al., 2021). Polycomb Repressive Complexes (PRCs) also play crucial roles in connecting histone modifications and DNA methylation in neurodevelopment (Loaeza-Loaeza et al., 2020).

This review aims to contextualize the distinct episignatures associated with pathogenic variants in the *TRIP12* and *USP7* genes within their respective roles in the ubiquitination pathway. Firstly, we will delve into the roles of these genes within the ubiquitination pathway. Secondly, we will explore how these roles may influence the episignature. Finally, we will investigate the interconnections between the functional roles of the proteins encoded by these genes and how such connections might account for the differing episignatures observed.

### Ubiquitination pathway

**Figure 1:**
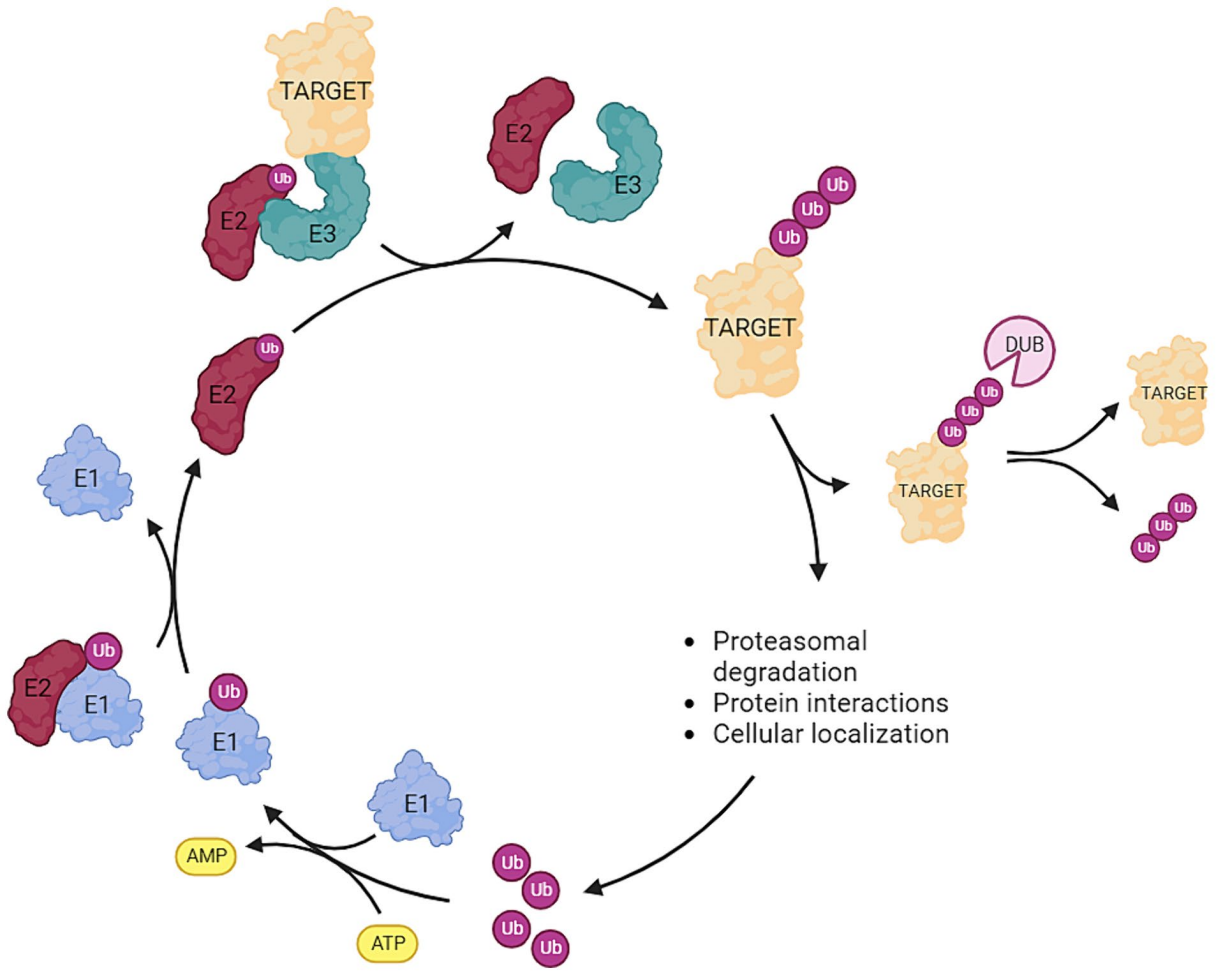
Overview of the ubiquitination pathway: Ubiquitin (ub) is attached to an E1 activating enzyme following ATP hydrolysis. Subsequently, ub is transferred to the E2 conjugating enzyme, and then the E3 ligase facilitates the transfer of ub from E2 to the target protein. Deubiquitinases (DUBs) are responsible for removing ubiquitin molecules from the target protein. The type (mono-, poly-) and the site of ubiquitination exert distinct effects on the fate of the target protein. Ubiquitination can serve as: (1) a marker for proteasomal degradation, (2) a modifier of protein interactions, or (3) a determinant of cellular localization (Created with BioRender).

Ubiquitin is a relatively small protein of approximately 8.6 kDa and is present in virtually all tissues of eukaryotic organisms. Ubiquitin serves as a key regulator in many distinct cellular mechanisms and is encoded by four genes in the human genome (Kimura and Tanaka, 2010). The regulatory process of ubiquitination is highly dynamic and involves the attachment or removal of one or multiple ubiquitin proteins to target proteins. This modification serves as a versatile cellular mechanism for protein degradation, cellular localization, and modulation of protein–protein interactions. The intricate interplay between E1, E2, and E3 enzymes ensures the specificity and diversity of ubiquitin-mediated cellular processes (Figure 1). Initially, a small ubiquitin protein forms a thioester linkage with an active site cysteine on an E1 activating enzyme following ATP hydrolysis. Subsequently, ubiquitin is transferred to the active site cysteine of E2 conjugating enzymes. Ultimately, E3 ligases facilitate the transfer of ubiquitin from E2 conjugating enzymes to the target protein through various mechanisms. The E3 ligase accomplishes this by utilizing the ubiquitin linked to the active site cysteine of its subunits—Homologous to E6AP C-Terminus (HECT) and RING-Between-RING (RBR). Once this process is completed, ubiquitin is transferred onto the target protein. The RING families of E3 enzymes function as scaffold proteins, aiding in the linkage between E2 and the target protein but is not directly involved in the transfer and bonding of the ubiquitin protein itself. It should be noted that the specific ubiquitin bond site, and whether one or multiple ubiquitin proteins are bonded, lead to diverse fates for the target protein. For example, K11-linked ubiquitin proteins are instrumental in regulating cell-cycle progression and cell division. K48, K63, and K11-linked ubiquitin proteins modify kinase activity in response to cellular stress. K63 ubiquitination is crucial for assembling protein complexes and assists in protein sorting during endocytosis and autophagy. Polyubiquitination of K11 or K48 marks the target protein for degradation by the proteasome (Ardley and Robinson, 2005; Rape, 2018; Doyle et al., 2022; Jolly et al., 2022). As aforementioned, ubiquitination processes are highly dynamic. Beyond the addition of ubiquitin molecules to proteins, opposing components of the ubiquitination machinery possess the capability to remove ubiquitin proteins from target proteins. These components, known as deubiquitinases (DUBs), play, among other roles, a critical role in preventing protein degradation. Specific DUBs are also pivotal in the reuse and recycling of ubiquitin proteins (Parihar and Bhatt, 2021; Liao et al., 2022). The fate of a ubiquitinated protein—whether it is recovered or degraded—depends on the type of ubiquitin linkage and the specific DUBs involved. K48-linked polyubiquitination typically signals for proteasomal degradation, while K63-linked polyubiquitination is often associated with non-degradative functions such as signaling. The activity and specificity of DUBs are controlled by various factors, including post-translational modifications, cellular localization, and interactions with other proteins. This dynamic regulation by DUBs ensures precise control over protein homeostasis, determining whether an ubiquitinated protein is destined for degradation or recovery (Clague et al., 2015; Parihar and Bhatt, 2021; Jolly et al., 2022; Liao et al., 2022).

### Ubiquitination processes during early development

Ubiquitination plays a crucial role in various diseases and disorders. To delve into its specific involvement in neurodevelopmental disorders, comprehending its role(s) in embryonic development is paramount. The anaphase-promoting complex (APC/C) is a crucial protein complex within the ubiquitination pathway, influencing cell proliferation and differentiation during development (Ardley and Robinson, 2005). In undifferentiated cells, APC/C, along with its co-factor Cell Division Cycle Protein 20 Homolog (CDC20), triggers the degradation of anaphase inhibitors and initiates sister chromatid segregation during mitosis, promoting cell division. Conversely, upon cell differentiation, APC/C collaborates with CDC20 Homolog 1 (CDH1), preventing cells from entering the S-phase and inhibiting cell division in differentiated cells by promoting the degradation of S-phase entry-inducing kinases. These observations highlight APC/C’s role in protein degradation as a recognized E3 ligase that marks its substrates for proteasomal degradation by ubiquitination. Additionally, APC/C directs cellular fate. In brain development, APC/C contributes significantly to synapse development, axon growth, and cell patterning within the cerebellar cortex. APC/C stands as the most comprehensively understood E3 ligase, playing a crucial role in neurodevelopment by regulating the degradation of proteins involved in neuronal migration, dendritic morphology, synaptic plasticity, and axon guidance pathways. By controlling protein turnover, APC/C ensures precise control essential for the formation and refinement of neural circuits in the cerebral cortex. However, other E3 ligases with similar functions have also been described (Huang et al., 2015; Rape, 2018). It is widely acknowledged that epigenetic modifications play a crucial role in cell differentiation, particularly during early development. Among these, the Polycomb repressive complexes (PRCs) are particularly significant in embryonic development. PRCs are divided into two main classes: PRC2 acts as the initiation complex, while PRC1 functions as the maintenance complex. These complexes establish connections between histone modifications and DNA methylation and interact with proteins such as USP7 and TRIP12. This suggests that PRCs may serve a pivotal role in linking the episignatures associated with USP7 and TRIP12-related NDDs to the function of these proteins (Fahrner and Bjornsson, 2014). Furthermore, it implies that these three proteins have essential functions during embryonic development, potentially explaining their association with NDDs.

### Interplay between histone modifications and DNA methylation

Although the relationship between histone modifications and DNA modifications is widely acknowledged within scientific consensus, the underlying molecular mechanism(s) that connect both complex epigenetic entities is not yet completely resolved. Notably, one of the first robust diagnostic implementable examples of a DNA methylation signature comprised a disorder linked to pathogenic variation within the *NSD1* gene, which encodes a histone H3 lysine 36 methyltransferase (Choufani et al., 2015). Similarly and for this review particularly, TRIP12 and USP7 are also known histone modifiers. Pathogenic variation in these genes were shown to represent sensitive and selective DNA methylation signature (van der Laan et al., 2022a, 2023). Again, while histone and DNA modifications unquestionably exert mutual influence, the exact underlying mechanism remains elusive. Nonetheless, several key proteins involved in this interplay have been previously identified.

Epigenetic modifications are widely acknowledged to hold significant importance in cell differentiation, particularly during early developmental stages. Specifically, the PRCs exert crucial influence during embryonic development. PRC2 acts as the initiation complex, while PRC1 serves as the maintenance complex (Brunet et al., 2020). These complexes are involved in linking histone modifications to DNA methylation and exhibit interactions with USP7 and TRIP12. This suggests a potential crucial role of PRCs in connecting the episignatures of USP7 and TRIP12 to their respective functions. Furthermore, the latter association implies that these proteins USP7, TRIP12, and the PRCs play indispensable roles during embryonic development, possibly elucidating their links to NDDs (Brunet et al., 2020; van der Laan et al., 2022a, 2023). A detailed exploration of the interaction between these proteins and PRCs will be discussed in subsequent sections of this review.

### USP7 and TRIP12

Pathogenic variants in *USP7* [also called Herpes virus-associated ubiquitin-specific protease 7 (HAUSP)] lead to Hao-Fountain Syndrome. This syndrome is characterized by intellectual disability, speech delay, behavioral abnormalities, autism spectrum disorder, seizures, hypogonadism, and mild dysmorphic features. The *USP7* gene, located on chromosome 16p13.2, encodes for a deubiquitinase, enabling the removal of ubiquitin molecules from target proteins (Hao et al., 2015; Fountain et al., 2019; van der Laan et al., 2023; Wimmer et al., 2024). Previously we showed that pathogenic variants in *USP7* exhibit with a specific and sensitive DNA methylation episignature (van der Laan et al., 2023). USP7 exists in several isoforms, including the well-characterized Isoform 1 (differs in the 5’ UTR and coding sequence compared to variant 1, encoding the longest isoform), Isoform 2 (has a shorter and distinct N-terminus compared to isoform 1), Isoform 3 (contains an alternate 5′ terminal exon and an additional internal exon, which results in translation initiation from an in-frame downstream start codon compared to variant 1, has a shorter N-terminus compared to isoform 1), and Isoform 4 (contains an alternate 5′ terminal exon, which results in translation initiation from an in-frame downstream start codon compared to variant 1, has a shorter N-terminus compared to isoform 1). Isoform 1, containing all known functional domains, is the predominant form expressed in both the brain and peripheral tissues. This isoform is crucial for the stabilization of key regulatory proteins such as p53 (USP7, 2024).

**Figure 2:**
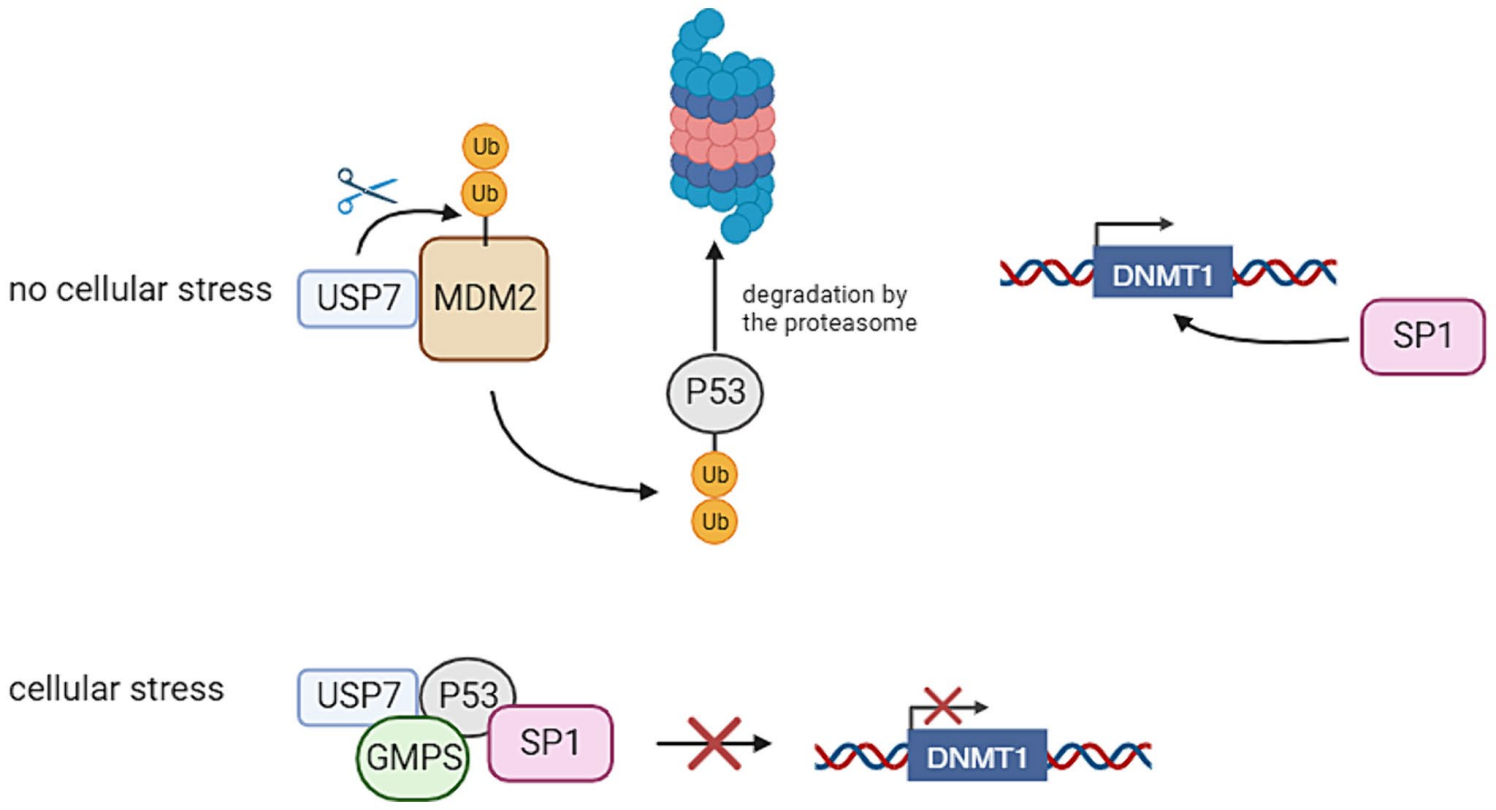
Ubiquitin Specific Peptidase 7 (USP7) regulates the stability of P53 and thereby the transcription of DNA Methyltransferase 1 (DNMT1). In the absence of cellular stress, USP7 has an interaction with Mouse Double Minute 2 Homolog 2 (MDM2), thereby stabilizing it. This leads to the degradation of P53, because this protein is ubiquitinated by MDM2. This allows Specificity Protein 1 (SP1) to bind to the DNMT1 promoter, thereby activating its transcription. If cellular stress is present, USP7 has an interaction with P53 through the interference of Guanosine Monophosphate Synthetase (GMPS). This has the result that P53 binds to SP1, preventing it from binding to the DNMT1 promoter. This inhibits the transcription of DNMT1 (Created with BioRender).

Within neuronal stem cells, USP7 plays a role in deubiquitinating key proteins such as Restrictive Element-1 Silencing Transcription Factor (REST), c-MYC, and Sex Determining Region Y (SRY)-Related High-Mobility Group-Box (SOX), preventing their proteasomal degradation and thereby maintaining stem cells in an undifferentiated state. Additionally, in hypothalamic neurons, USP7 safeguards Tripartite Motif Containing 27 (TRIM27) from degradation, impeding the delivery and degradation of endosomal cargo at the lysosome. Notably, pathogenic variants in the gene encoding Melanoma Antigen-L2 (MAGE-L2), a protein complexed with TRIM27, have associations with various neurodevelopmental disorders (Wertz and Murray, 2019). Moreover, the absence of USP7 in mouse brain results in elevated levels of P53, leading to apoptosis and reduced brain size. USP7 regulates P53 levels by preventing the degradation of P53 E3 ligase Mouse Double Minute 2 Homolog 2 (MDM2) (Jolly et al., 2022). Figure 2 provides an overview of the functions of *USP7*. The significance of USP7 in relation to the PRC will also be highlighted in this review. It should be noted that as of yet, the connection of these functions with DNA methylation remains unclear. This gap in understanding may provide a crucial avenue for further investigation into the intricate regulatory mechanisms governing neuronal development.

**Figure 3:**
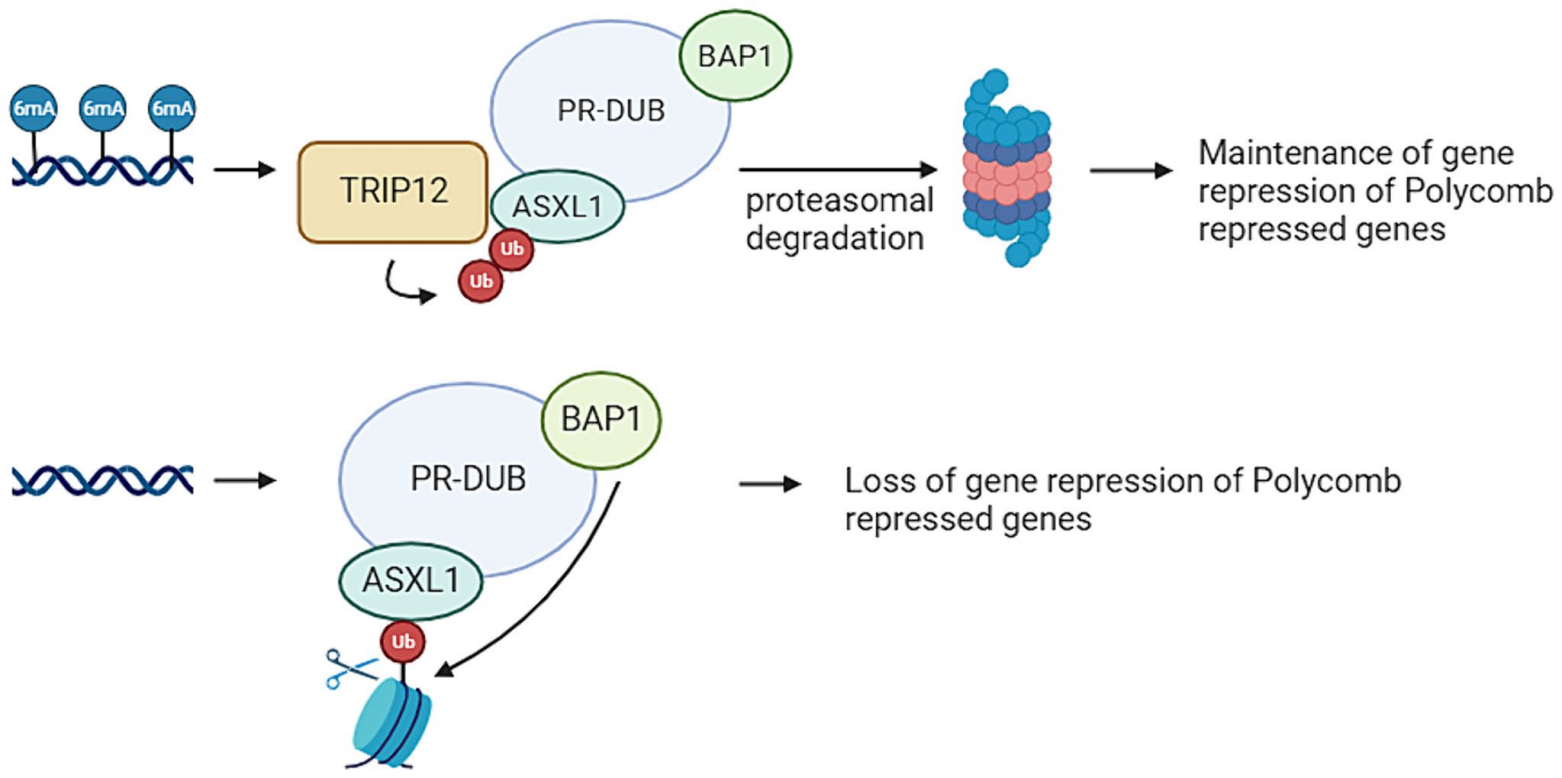
Thyroid Hormone Receptor Interactor 12 (TRIP12) mediates the maintenances of a repressive ubiquitin signal added by the Polycomb Repressive Complex (PRC). PRC mediated DNA methylation on adenine position 6 (6 mA) results in TRIP12 polyubiquitination of Additional Sex Combs Like 1 (ASXL1), which is a subunit from the Polycomb Repressive Deubiquitinase (PR-DUB) complex. This leads to the proteasomal degradation of PR-DUB and therefore the maintenance of the ubiquitin marks added by PRC1. If no 6 mA is present on PRC repressed genes, there is also no TRIP12 present. This means ASXL1 can target PR-DUB to H2AK119 monoubiquitination. This leads of the deubiquitination of that histone by BRCA1 Associated Protein 1 (BAP1) and therefore a loss of the repressive mark (Created with BioRender).

*TRIP12* is a gene that plays an opposite role in the ubiquitination pathway compared to *USP7*. Situated on chromosome 2q36.3, TRIP12 encodes an E3 ligase (Brunet et al., 2020). Figure 3 provides an overview of the functions of *TRIP12.* Pathogenic variants within the *TRIP12* gene have been linked to various disease patterns, including cancer and neurodegenerative diseases. In addition, Clark-Baraitser syndrome is a neurodevelopmental disorder also linked to pathogenic variations within *TRIP12*. Symptoms of this syndrome include intellectual disability, and sometimes autism spectrum disorders, speech delay, obesity and dysmorphic features like narrow up-slanting palpebral fissures and a distinct mouth with downturned corners (Aerden et al., 2022; van der Laan et al., 2022a). Previous studies have indicated that pathogenic variants in *TRIP12* correlate with a specific and distinctive DNA methylation signature. Notably, a considerable portion (22%) of differentially methylated probes in Clark-Baraitser syndrome exhibit overlap with the DNA methylation signature associated with *USP7*, albeit with an opposing effect. Specifically, *TRIP12* demonstrates primarily hypomethylation, whereas *USP7* exhibits predominantly hypermethylation. Given that TRIP12 functions in ubiquitination and USP7 in deubiquitination, this contrasting methylation pattern suggests their potential opposite roles, aligning with observations in the episignature (van der Laan et al., 2022a).

TRIP12, also has multiple isoforms (26), notably Isoform 1 is the dominant form, playing a vital role in protein degradation and DNA damage response, which are essential for neuronal function (TRIP12, 2024). Since there are not many studies about different isoforms in comparison with clinical presentations, isoform-specific studies are important to understand those disease mechanisms.

The examination and comparison of phenotypic characteristics between patients harboring pathogenic variations in *USP7* (Hao-Fountain syndrome) and *TRIP12* (Clark-Baraitser syndrome) genes reveal intriguing distinctions and commonalities in clinical presentations, particularly in terms of facial features and additional associated medical issues. HAFOUS is commonly associated with seizures and structural brain abnormalities, where CLABARS may present other features such as obesity (Fountain et al., 2019; Aerden et al., 2022; Wimmer et al., 2024). Both *USP7* and *TRIP12* pathogenic variations have been associated with a spectrum of neurological and developmental abnormalities, sharing overlapping features such as intellectual disability, developmental delay, motor delay, speech delay, facial dysmorphisms, and growth abnormalities. Understanding these observable variations and shared phenotypic traits between these syndromes is critical for accurate clinical diagnoses, tailored management strategies, and potentially unraveling the distinct pathophysiological mechanisms underlying each condition. Moreover, these findings pave the way for further research into the intricate genetic interplay contributing to the diverse clinical manifestations observed in individuals affected by *USP7* and *TRIP12* pathogenic variations.

### Knock out models

Mouse models have been crucial in unraveling the intricate roles of TRIP12 and USP7 genes in neurodevelopment and embryogenesis, shedding light on their potential implications in various biological conditions, particularly autism spectrum disorders in the case of USP7. Studies utilizing mouse models carrying pathogenic variations or alterations in these genes have revealed compelling insights into their functions.

The USP7 gene, implicated in autism spectrum disorders, has been investigated in mouse models to comprehend its involvement in neurodevelopment. Brain-specific overexpression of USP7 in primary mouse neurons led to an increase in dendritic branching and length (Qiao et al., 2022), while a brain-specific knockdown resulted in opposing alterations, underscoring USP7’S role in regulating dendritic morphology (Kon et al., 2011). Additionally, USP7 was found to deubiquitinate the X-linked inhibitor of apoptosis protein (XIAP), influencing caspase 3 activity and dendritic pruning. Introducing USP7 into the brains of prenatal mice resulted in abnormal neuron migration and increased dendritic arborization (Qiao et al., 2022). Notably, injecting AAV-USP7 in neonatal overexpression mice induced autism spectrum disorder-like behaviors including aberrant social interactions, repetitive behaviors, as well as changes in somatosensory sensitivity, emphasizing the potential link between USP7 dysregulation and autism spectrum disorders phenotypes (Qiao et al., 2022).

Conversely, investigations into Trip12, a HECT-type E3 ubiquitin ligase, unveiled its critical role in mouse embryogenesis. Mouse models with a homozygous pathogenic variation disrupting TRIP12’s ubiquitin ligase activity displayed embryonic lethality during mid-development due to growth arrest and increased expression of the cell cycle regulator p16. Notably, heterozygous models of TRIP12 deficiency have also shown phenotypes, including developmental delay and abnormal placental morphology. However, TRIP12 mutant embryonic stem cells (ESCs) remained viable, showing decreased proliferation but retaining undifferentiated states and differentiation capabilities. These TRIP12 mutant ESCs exhibited altered gene expression patterns and increased levels of BAF57, a component of the SWI/SNF chromatin remodeling complex. This suggests that TRIP12 is crucial for global gene expression and plays a pivotal role in regulating mouse development (Kajiro et al., 2011).

These mouse models, illuminating the roles of USP7 and TRIP12, offer invaluable insights into their functions in neurodevelopment, embryogenesis, and gene regulation. They are crucial for investigating how USP7 and TRIP12 gene variations affect episignatures by allowing researchers to manipulate gene expression and observe resulting changes in DNA methylation patterns. By studying these models, we can understand how altered USP7 and TRIP12 activity influences chromatin remodeling and DNA methylation, crucial for neurodevelopmental disorders and providing insights into disease mechanisms and potential therapeutic targets.

## Conclusion

The ubiquitination pathway is instrumental in regulating protein stability, localization, and interactions, pivotal not only in neurodevelopment but also in embryonic development, immunity, and cancer. This pathway likely intertwines these aspects. Numerous genes linked to an episignature are involved in the ubiquitination pathway, suggesting a link between ubiquitination and DNA methylation. Our review indicates the ubiquitination pathway’s crucial role in histone modifications. However, the intricate interplay between histone modifications and DNA methylation remains largely unknown, posing challenges in establishing a direct connection between the episignature and the role of ubiquitination enzymes within this pathway.

In conclusion, while we cannot definitively explain how pathogenic variants in *TRIP12* and *USP7* result in specific DNA methylation signatures, correlations exist between the functions of these genes’ protein products and DNA methylation. Further research is warranted to elucidate the intricate relationship between the ubiquitination pathway, histone modifications, and DNA methylation for a comprehensive understanding of their combined roles in biological processes.

### Future perspectives and experiments

As discussed earlier, the continual discovery of new Neurodevelopmental Disorder (NDD)-associated episignatures leads to increased overlap in DMPs among different syndromes. This overlap presents a potential challenge for future diagnoses. Addressing this challenge requires understanding the pathogenic variation’s specific impact on DNA methylation patterns. This review represents an initial step in that direction, providing an overview based on current knowledge. However, further investigations are imperative to better comprehend the intricate connections between histone modifications, DNA methylation, and how alterations in the ubiquitination pathway contribute to abnormal DNA methylation signatures.

A significant challenge in collecting samples from NDD patients is the inability to obtain brain cells from living individuals. Consequently, blood samples are often utilized, and their DNA methylation patterns are analyzed. While several studies demonstrate a high degree of similarity between brain and blood cell DNA methylation patterns, it is important to note that similarity does not equate to identity. Moreover, the loss of a specific protein may yield diverse effects in different cell types (Tylee et al., 2013; Younesian et al., 2022).

As discussed earlier, there are other genes involved in the ubiquitination pathway that already possess episignatures, as well as genes whose functions suggest an, as-yet-undiscovered episignature. We can categorize these genes into five distinct categories:

1. *UBA1*, this gene encodes ubiquitin-activating enzyme E1, which initiates the ubiquitination cascade by activating ubiquitin in an ATP-dependent manner (Sakuma et al., 2023).
2. *UBE2* family, genes encoding ubiquitin-conjugating enzymes (E2) such as *UBE2D*, *UBE2E*, *UBE2N*, and others. These enzymes work with E1 enzymes to transfer activated ubiquitin to the target protein (Zhang and Yang, 2022).
3. E3 ubiquitin ligases, there are hundreds of genes encoding E3 ligases that facilitate the transfer of ubiquitin from E2 enzymes to specific protein substrates. Examples include: *CUL3, CUL4A, CUL4B, CUL5*: These are members of the cullin family and form complexes with other proteins to create E3 ubiquitin ligases (Hu et al., 2004). *MDM2, SKP1, FBXW7, PARK2*: These are examples of genes encoding E3 ligases that have specific substrates and functions in ubiquitination (Ardley and Robinson, 2005).
4. DUBs, these genes encode enzymes that remove ubiquitin from proteins and thereby reverse the process of ubiquitination. Examples include ubiquitin-specific protease (USP) family genes such as *USP7*, *USP8*, etc., and other families like ovarian tumor (OTU) and ubiquitin carboxyl-terminal hydrolase (UCH) (Chen et al., 2021; Min et al., 2023).
5. Ubiquitin itself. While not a gene directly involved in the ubiquitination process, the genes encoding ubiquitin (UBB and UBC) produce the ubiquitin protein that becomes attached to target proteins (Rape, 2018).

Some of the above mentioned genes have been discussed in relation to episignatures, while for most of them, no episignature has been identified yet. This absence could potentially be attributed to the novelty of research regarding DNA methylation episignatures, indicating that investigations into these specific patterns of methylation in these genes might be relatively recent or still ongoing.

## Author contributions

LL: Conceptualization, Project administration, Validation, Visualization, Writing – original draft, Writing – review & editing. NV: Conceptualization, Visualization, Writing – original draft. MM: Supervision, Writing – review & editing. PH: Project administration, Supervision, Writing – review & editing.
